# Risk factors for instent restenosis of sirolimus-coated stents in coronary intervention for patients with unstable angina

**DOI:** 10.1038/s41598-024-52567-6

**Published:** 2024-01-30

**Authors:** Dongchao Liu, Zheng Xue, Jingxian Qi, Liang Yin, Bing Duan, Lin Wu, Kun Yang, Bulang Gao, Qinying Cao, Jie Mi

**Affiliations:** Department of Cardiology, Shijiazhuang People’s Hospital, 365 South Jianhua Street, Shijiazhuang, 050011 Hebei China

**Keywords:** Cardiology, Diseases, Medical research, Risk factors

## Abstract

To investigate the instent restenosis rate of sirolimus-coated stents in percutaneous coronary intervention (PCI) and risk factors for in-stent restenosis, patients with unstable angina (UA) caused by coronary artery stenosis were enrolled, and all clinical and imaging data were analyzed. Among 143 enrolled patients with UA aged 35–83 (mean 60.9 ± 10.0) years enrolled, there were 114 (79.7%) male and 29 (20.3%) female patients. Arterial stenosis was present in one coronary artery in 6 (4.2%) patients, in two coronary arteries in 20 (14.0%) patients, in three arteries in 116 (81.1%), and in four coronary arteries in 1 (0.7%) patient. Stenting was successfully performed in all (100%) patients, and 181 stents were deployed. The quantitative flow ratio (QFR) was 0.92 ± 0.03 (range 0.84–0.96) immediately after stenting, and the TIMI was grade 3 in all patients. The diameter of the stents deployed ranged 2.25–4 mm (mean 3.04 ± 0.44) with a length ranging 10 mm to 104 mm (mean 32.73 ± 15.5). Follow-up angiography was performed in all patients with a duration of 1–92 (mean 15.0 ± 18.8) months. Instent restenosis ≥ 50% occurred in 25 (17.5%) patients. In univariate logistic regression analysis, significant (P < 0.05) risk factors for instent restenosis ≥ 50% were QFR (OR 0.036, 95% CI 0.13–0.97), stent diameter (OR 0.43, 95% CI 0.18–0.92), hypertension (OR 3.16, 95% CI 1.02–9.82), smoking (OR 0.31, 95% CI 0.11–0.89), and neutrophil count (OR 2.22, 95% CI 1.10–5.44). In multivariate analysis, QFR (OR 0.02, 95% CI 0.002–0.19), stent diameter (OR 0.06, 95% CI 0.005–0.59), hypertension (OR 6.75, 95% CI 1.83–35.72) and neutrophil count (OR 276.07, 95% CI 12.32–10,959.95) were significant (P < 0.05) independent risk factors for instent restenosis ≥ 50%. In conclusion, certain instent restenosis rates occurs after the sirolimus-eluted coronary stent deployment for the treatment of coronary artery stenosis in patients with UA, and quantitative flow ratio after stenting, stent diameter, hypertension, and neutrophil count are significant risk factors for instent restenosis of the sirolimus-coated stents in coronary intervention.

## Introduction

With ageing and continuous development of life quality, the prevalence of cardiovascular disease is on the rise, so is the mortality of cardiovascular disease^1–3^. Coronary heart disease contributes a great deal to the mortality of cardiovascular disease, from 8.6% in 1990 to 15.2% in 2013^4^. It is thus important to prevent and treat coronary heart disease. Unstable angina (UA) is caused by coronary artery plaque and stenosis, and timely treatment of patients with UA is effective in preventing severe consequences. Percutaneous coronary intervention (PCI) is one important approach for the treatment of coronary artery stenosis. Drug-eluting stents such as sirolimus-coated or sirolimus-eluting coronary stents have been applied in PCI for the treatment of coronary artery stenosis and have achieved great effects^5–9^. With years of research, the pathogenesis and characteristics of instent restenosis have been revealed, and the intra-coronary imaging technology has further elucidated the mechanism and potential basis of instent restenosis^10–12^. However, despite substantial improvements in the drug-eluting stent technologies, instent restenosis still occurs at a rate of 1–2% per year with the contemporary drug-eluting stent platforms, requiring target lesion revascularization^13^. More indicators that can predict the occurrence of instent restenosis in patients receiving drug-eluting stents are needed for further investigation. This study was consequently performed to investigate the instent restenosis rate of sirolimus-coated coronary stents and the relevant risk factors for instent restenosis in the treatment of patients with UA.

## Materials and methods

### Subjects

This single-center retrospective study was performed between December 2014 and May 2022 after approval by the ethics committee of Shijiazhuang People’s Hospital, and the signed informed consent was waived because of the retrospective study design. All methods were performed in accordance with the relevant guidelines and regulations. Patients with UA confirmed to be caused by coronary artery stenosis and treated with percutaneous coronary intervention (PCI) were enrolled. The inclusion criteria were patients with UA-related chest tightness and pain in the anterior chest area caused by coronary artery stenosis which was confirmed by coronary artery angiography and treated with selective PCI 2–3 days after admission. Patients with coronary artery stenosis and acute myocardial infarction treated emergently were not enrolled.

### Intervention

Before PCI, dual antiplatelet therapy with aspirin 100 mg once per day and clopidogrel 75 mg once daily (or aspirin 100 mg once per day and ticagrelor 90 mg twice per day) was conducted in all patients. Coronary intervention was performed firstly with balloon expansion of the stenosis followed by deployment of one or multiple stents in series for long-segment stenosis. After stenting, dual antiplatelet therapy was continued with aspirin 100 mg once per day and clopidogrel 75 mg once daily for at least 12 months or a longer time. Atorvastatin 20 mg once per night or rosuvastatin 10 mg once per night was administered for every patient for lifetime.

### Data evaluation

The following data were collected and analyzed: the patient’ sex, age, symptoms, coronary arteries with stenosis, stenotic degree, stent deployment, number of stent deployed, location of stenting, stent diameter, length of the stented segment of artery, post-stenting dilation, quantitative flow ratio (QFR) and residual stenosis after stenting, Thrombolysis in Myocardial Infarction (TIMI)^14^, follow-up duration, instent restenosis, dual antiplatelet therapy, hypertension, diabetes mellitus, smoking, low and high density lipoprotein, total cholesterol (TC), triglycerides (TG), neutrophil count, lymphocyte count, neutrophil/lymphocyte ratio (NLR), platelet count, uric acid, and homocysteine. Instent restenosis was defined as stenosis of the coronary artery diameter ≥ 50%, including the stented coronary artery and arterial segment within 5 mm from the edge of the stent^15^. Two physicians who were blinded to the clinical data analyzed the data, and if in disagreement, a third senior physician was involved to reach an agreement.

### Statistical analysis

The statistical analysis was performed with the SPSS version 22.0 software (IBM, Chicago, IL, USA). Continuous measurement data were presented in means and standard deviation if in the normal distribution and tested with the t-test or median and interquartile range and tested with the Mann–Whitney *U* test if not in the normal distribution. Categorical data were presented as frequency and percentage and tested with the Chi square test. The univariate logistic regression analysis was performed for risk factors for instent restenosis. Factors significant in the univariate analysis were entered into the multivariate logistic regression analysis for independent risk factors for instent restenosis. The Odds ratio (OR) with 95% confidence interval (CI) was calculated, and the receiver operating characteristic (ROC) curve analysis was performed to investigate the continuous measurement data, with calculation of the following parameters: the area under the ROC curve (AUC), sensitivity, specificity, positive predictive value (PPV), and negative predictive value (NPV). The Kaplan–Meier curve was plotted with the time from stent implantation to instent restenosis. The statistical significant P was set at < 0.05.

## Results

A total of 143 patients with UA (Unstable angina) aged 35–83 (mean 60.9 ± 10.0) years were enrolled, including 114 (79.7%) male and 29 (20.3%) female patients (Table 1). At admission to the hospital, hypertension was present in 95 (66.4%) patients, diabetes mellitus in 48 (33.6%), and smoking in 57 (39.9%). In laboratory test, the low density lipoprotein was 1.38–4.56 (mean 2.67 ± 0.67) mmol/L, total cholesterol (TC) 1.76–5.80 (3.28 ± 0.80) mmol/L, triglycerides (TG) 0.36–8.4 (1.47 ± 1.10) mmol/L, high density lipoprotein 0.54–3.17 (0.99 ± 0.38) mmol/L, neutrophil count 4.02 ± 1.29 (1.77–9.7) × 10^9^/L, lymphocyte count 1.53 ± 0.45 (0.68–2.9) × 10^9^/L, neutrophil/lymphocyte ratio (NLR) 2.97 ± 1.47 (1.1–12.1), platelet count 219.56 ± 68.56 (107–540) × 10^9^/L, uric acid 263.02 ± 94.35 (202–600) μmol/L, and homocysteine 16.9 ± 2.53 (13.2–19.6) μmol/L. At the initial diagnosis, arterial stenosis was present in one coronary artery in 6 (4.2%) patients, in two coronary arteries in 20 (14.0%) patients, in three arteries in 116 (81.1%), and in four coronary arteries in 1 (0.7%) patient. The stenosis was present in the left main coronary artery in 13 (9.1%) patients.

**Table 1:** Clinical data and treatment.

Variables	Data
Patients data	
No. of patients	143
F/M	29/114
Age (y)	60.9 ± 10.0 (35–83)
Co-morbidities	
Hypertension (n, %)	95 (66.4%)
Diabetes mellitus (n, %)	48 (33.6%)
Smoking (n, %)	57 (39.9%)
Laboratory test	
TC (mmol/L)	1.76–5.80 (3.28 ± 0.80)
TG (mmol/L)	0.36–8.4 (1.47 ± 1.10)
High density lipoprotein (mmol/L)	0.54–3.17 (0.99 ± 0.38)
Neutrophil count (× 10^9^/L)	4.02 ± 1.29 (1.77–9.7)
Lymphocyte count (× 10^9^/L)	1.53 ± 0.45 (0.68–2.9)
Neutrophil/lymphocyte ratio	2.97 ± 1.47 (1.1–12.1)
Platelet count (× 10^9^/L)	219.56 ± 68.56 (107–540)
Uric acid (μmol/L)	263.02 ± 94.35 (202–600)
Homocysteine (μmol/L)	16.9 ± 2.53 (13.2–19.6)
Platelet count	219.56 ± 68.56 (107–540)
Coronary artery stenosis	
In one artery (n, %)	6 (4.2%)
In two arteries (n, %)	20 (14.%)
In 3 arteries (n, %)	116 (81.1%)
Stenting location	
Left main trunk	7 (4.9%)
Left anterior descending artery	58 (40.56%)
LCX	32 (22.38%)
Right coronary artery	51 (35.7%)
After stenting	
QFR	0.92 ± 0.03 (0.84–0.96)
TIMI grade	3
Stent diameter (mm)	3.04 ± 0.44 (2.25–4)
Stented length (mm)	32.73 ± 15.5 (10–104)
Post-stenting dilation (n, %)	53 (37.1%)
No. of stents	181
Patients receiving 1 stent	108 (75.5%)
Patients receiving 2 stents	32 (22.4%)
Patients receiving 3 stents	3 (2.1%)
Follow-up	
Follow-up duration (m)	15.0 ± 18.8 (1–92)
No. of patients (n, %)	143 (100%)
Instent stenosis ≥ 50% (n, %)	25 (17.5%)
Retreatment (n, %)	
Drug-balloon expansion	7 (28%)
Stenting	5 (20%)
Balloon expansion only	1 (5.9%)

*QFR* quantitative flow ratio, *TC* total cholesterol, *TG* triglycerides, *TIMI* thrombolysis in myocardial infarction.

Selective PCI was performed 2–3 days after admission. Stents were successfully deployed in all (100%) patients, and 181 sirolimus-coated stents were deployed in 143 patients, with one stent in 108 (75.5%) patients, 2 stents in 32 (22.4%), and 3 stents in 3 (2.1%). The stent was deployed in the left main trunk before branching in 7 (4.9%) patients, left anterior descending artery in 58 (40.56%) patients, LCX (left circumflex artery) in 32 (22.38%), and right coronary artery in 51 (35.7%). The QFR was 0.92 ± 0.03 (range 0.84–0.96) immediately after stenting, and the TIMI was grade 3 in all patients. The diameter of the stents deployed ranged 2.25–4 mm (mean 3.04 ± 0.44), and the length ranged 10–104 mm (mean 32.73 ± 15.5). Post-dilation was performed in 53 (37.1%) patients with a mean dilation pressure 15.2 ± 2.64 atmos.

**Figure 1:**
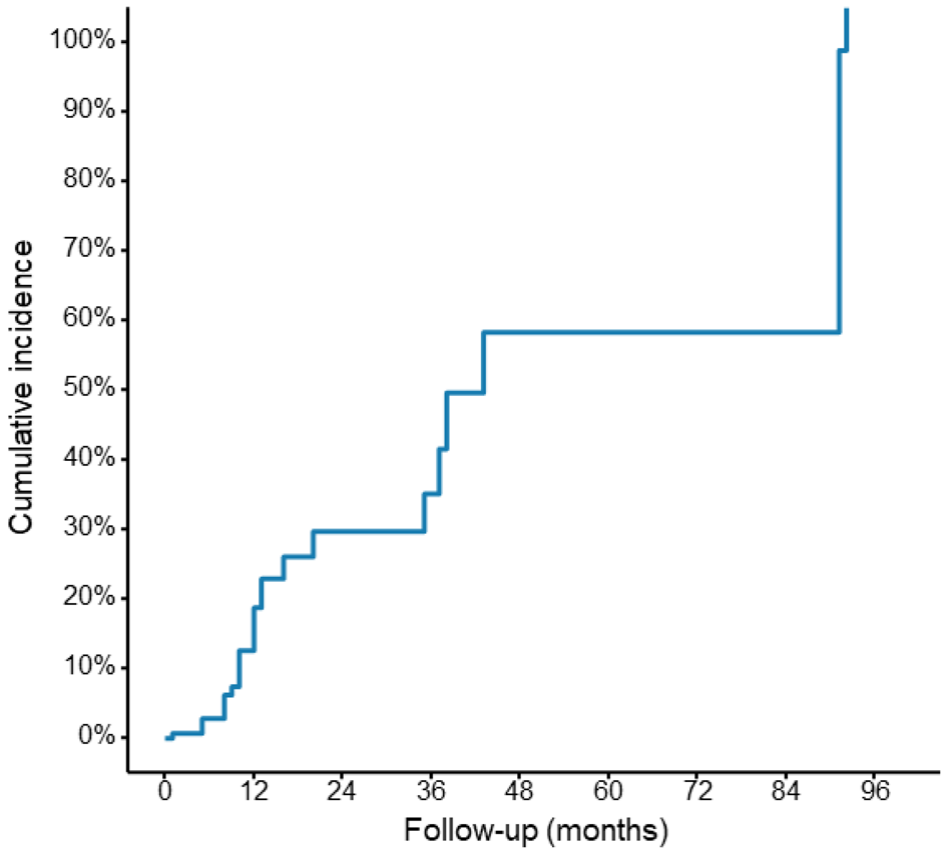
The Kaplan–Meier curve was plotted from stent implantation to instent restenosis at angiographic follow-up.

Follow-up angiography was performed in all patients with a duration of 1–92 (mean 15.0 ± 18.8) months. Instent restenosis ≥ 50% occurred in 25 (17.5%) patients, among whom, sirolimus-coated balloon expansion was performed in 7 (28%) patients, retreatment with stenting in 5 (20%), and percutaneous transluminal coronary angioplasty (PTCA) alone in 1 (5.9%). The Kaplan–Meier curve was plotted with the time from stent implantation to instent restenosis (Fig. 1).

In univariate logistic regression analysis of the following factors of sex, age, follow-up duration, number of stenosed coronary arterial branches, stenotic degree before treatment, location of stenting, stent diameter, stent length, post-dilation, dual antiplatelet therapy, hypertension, diabetes mellitus, smoking, low and high density lipoprotein, TC, TG, neutrophil and lymphocyte counts, platelet count, normal blood fat, NLR, uric acid, and homocysteine, significant (P < 0.05) risk factors for instent restenosis ≥ 50% were QFR (OR 0.036, 95% CI 0.13–0.97), stent diameter (OR 0.43, 95% CI 0.18–0.92), hypertension (OR 3.16, 95% CI 1.02–9.82), smoking (OR 0.31, 95% CI 0.11–0.89), and neutrophil count (OR 2.22, 95% CI 1.10–5.44). In multivariate analysis of the parameters significant in the univariate analysis, QFR (OR 0.02, 95% CI 0.002–0.19), stent diameter (OR 0.06, 95% CI 0.005–0.59), hypertension (OR 6.75, 95% CI 1.83–35.72) and neutrophil count (OR 276.07, 95% CI 12.32–10,959.95) were significant (P < 0.05) independent risk factors for instent restenosis ≥ 50%.

In sex stratification analysis, right coronary artery stenting (OR 0.27, 95% CI 0.07–0.99), QFR (OR 0.29, 95% CI 0.09–0.90), smoking (OR 0.33, 95% CI 0.11–0.98), neutrophil count (OR 2.57, 95% CI 1.24–7.42), and NLR (OR 2.75, 95% CI 1.12–7.66) were significant (P < 0.05) risk factors in males while uric acid was a significant (P < 0.05) risk factor in females for instent restenosis in the univariate analysis (Table 2). In the multivariate analysis using the significant factors in the univariate analysis, right coronary stenting (OR 0.17 95% CI 0.03–0.77) and smoking (OR 0.21, 95% CI 0.04–0.83) were significant (P < 0.05) independent risk factors for instent restenosis in males, but there were no independent risk factors in females (Table 2).

**Table 2:** Univariate and multivariate analysis for risk factor analysis of instent restenosis.

Variables	Univariate analysis			Multivariate analysis		
	Statistical value	P	OR (95% CI)	Statistical value	P	OR (95% CI)
Total						
QFR	12.22	0.0005	0.036 (0.13–0.97)	12.91	0.0003	0.02 (0.002–0.19)
Stent diameter	4.89	0.027	0.43 (0.18–0.92)	5.56	0.014	0.06 (0.005–0.59)
Hypertension	4.76	0.029	3.16 (1.02–9.82)	5.46	0.003	6.75 (1.83–35.72)
Smoking	5.53	0.019	0.31 (0.11–0.89)			
Neutrophil count	6.29	0.012	2.22 (1.10–5.44)	11.0	0.0009	276.07 (12.32–10,959.95)
Male						
RCA stenting	4.8	0.048	0.27 (0.07–0.99)	4.55	0.033	0.17 (0.03–0.77)
QFR	9.38	0.042	0.29 (0.09–0.90)			
Smoking	4.35	0.037	0.33 (0.11–0.98)	4.32	0.038	0.21 (0.04–0.83)
Neutrophil count	8.67	0.003	2.57 (1.24–7.42)			
NLR	4.12	0.04	2.75 (1.12–7.66)			
Female						
Uric acid	4.60	0.03	8 (1.17–54.72)			

*QFR* quantitative flow ratio, *OR* odds, ratio, *CI* confidence interval, *RCA* right coronary artery.

ROC curve analysis was performed for continuous measurement data for instent restenosis (Table 3). The AUC was 0.726 for QFR with a cutoff value of 0.90, sensitivity 0.76, specificity 0.693, PPV 0.345, and NPV 0.931. The stent diameter had an AUC 0.630 and a cutoff value 2.90 mm, and the neutrophil count had an AUC 0.611 and a cutoff value 4.4 × 10^9^/L in all patients. The AUC was 0.713, 0.662 and 0.578 for QFR, neutrophil count, and NLR, respectively, for male patients, and 0.738 for uric acid for women (Table 3).

**Table 3:** ROC curve analysis of continuous risk factors.

Variables	AUC	Cutoff	Sensitivity	Specificity	PPV	NPV
Total patients						
QFR	0.726	0.90	0.76	0.693	0.345	0.931
Stent diameter (mm)	0.630	2.90	0.56	0.65	0.255	0.874
Neutrophil count (× 10^9^/L)	0.611	4.4	0.60	0.693	0.273	0.890
Male						
QFR	0.713	0.90	0.722	0.687	0.302	0.930
Neutrophil count (× 10^9^/L)	0.662	4.40	0.667	0.698	0.293	0.918
NLR	0.578	2.9	0.556	0.698	0.256	0.893
Female						
Uric acid (μmol/L)	0.738	388.0	0.714	0.762	0.50	0.867

*ROC* receiver operating characteristics, *AUC* area under the ROC curve, *QFR* quantitative flow ratio, *PPV* positive predictive value, *NPV* negative predictive value, *NLR* neutrophil to lymphocyte ration.

## Discussion

In investigating the risk factors for instent restenosis of PCI in patients with UA, it was found that certain instent restenosis rates occurred after the sirolimus-eluting coronary stents deployment, and QFR after stenting, stent diameter, hypertension, and neutrophil count were significant risk factors for instent restenosis of sirolimus-coated stents in coronary intervention for patients with UA.

Patients enrolled in our study were those with unstable angina pectoris only, and no patients with acute myocardial infarction were included. Firstly, because of the severity, potential hemodynamic instability, and oxidative stress reactions in myocardial infarction, there may be more confounding risk factors to affect instent restenosis. Secondly, acute myocardial infarction may cause a heavy thrombus load on the coronary arteries. After stent implantation, there may be slow flow or no flow in the coronary arteries, especially after balloon dilation for complete deployment of the stent, which can affect instent restenosis. Moreover, emergent treatment of acute myocardial infarction may affect the performance of endovascular or other appropriate therapeutic approaches. Therefore, patients with acute myocardial infarction were excluded from this study.

Narrowing of the stented arterial segment greater than 50% of the arterial diameter is defined as instent restenosis. Instent restenosis is featured by progressive stenosis of a coronary artery after stenting and is caused by stent-induced mechanical injury to the arterial wall^16,17^. Balloon expansion and stenting in PCI will cause injury to the stented artery, which will further trigger an inflammatory response, resulting in an increase in circulating biomarkers like amyloid A, fibrinogen and C-reactive protein^16–19^. In addition, injury to the artery will activate the proliferation and migration of smooth muscle cells to produce extracellular matrix and final formation of neointimal tissue which may cause restenosis in case of excessive hyperplasia^16,20^.

Risk factors for instent restenosis have been divided into patient-related and stent-related ones^16^. In stent-related factors, implantation of a bare metal stent may cause a 30% rate of instent restenosis, and implantation of a second-generation drug-eluting stent has significantly decreased the instent restenosis rate to 12%^21^. Application of the first-generation drug-eluting stents compared to bare metal stents (OR 0.35) and use of the second-generation drug-eluting stents compared to the first-generation drug-eluting ones (OR 0.67) have been found to be independent predictors of lower instent restenosis rates in the follow-up duration of 6–8 months after stenting^16,21^. The type of implanted stent has been confirmed to be a strong predictor of instent restenosis^16,22^. Late healing combined with local inflammatory reactions and hypersensitivity reactions to the polymer and drug has become new causes of instent restenosis in spite of continuous progress in the stent platform^11,23^. Thinner stent struts have been associated with fewer clinical and angiographic restenosis events^24^, which indicates that stent design is a key factor for restenosis.

In our study, post-dilation was performed in 53 (37.1%) patients with a mean dilation pressure 15.2 ± 2.64 atmos. This rate of post-dilation was low. All patients in this study were treated by an experienced physician with many years of PCI experience in a large number of cases. When the stent was deployed, it often was given more than the nominal pressure to fully dilate the stent. After the stent was evaluated to be fully dilated and deployed on quantitative coronary angiography from at least two angles of projection, no further post-dilation was necessary. For a few cases in which the stent was not fully dilated, further post-dilation was performed, which is why the post-dilation rate was low. This may also contribute to decreases in arterial injury, hyperplasia, and instent restenosis in the stented segment.

In our study, stent diameter was a significant independent predictor for instent restenosis. A stent with a bigger diameter will well maintain the vascular lumen and will not create a lot of intimal hyperplasia to affect the arterial lumen. Moreover, QFR after stenting was also a significant independent predictor for instent restenosis. The QFR was 0.92 ± 0.03 (range 0.84–0.96) immediately after stenting in our study, and was a significant (P < 0.05) independent risk factor for instent restenosis with an AUC of 0.726 and a cutoff value of 0.90 in the ROC analysis. This was similar to that in the HAWKEYE study by Biscaglia et al.^25^. In the HAWKEYE study, seven hundred fifty-one vessels in 602 patients were analyzed, and after correction for potential confounding factors, post-PCI QFR ≤ 0.89 was associated with a threefold increase in the risk for the vessel-oriented composite endpoint.

The greater the QFR, the less frequent the instent restenosis. The QFR is associated with the stent diameter. Bigger stents will cause greater QFR after being fully expanded and deployed. Incomplete dilation of the stent for deployment is also found as a significant predictor of instent restenosis^16,26,27^. Incomplete dilation of the stent will result in a decreased QFR, flow compromise, late formation of intimal hyperplasia, and subsequent stenosis. Length > 20 mm (OR 3.06) of stents deployed has been found as a significant risk factor for instent restenosis^1^, however, the stent length was not found as a significant risk factor for restenosis in our study. This is probably because our study focused on the restenosis of sirolimus-coated stents whereas the relevant study^1^ dealt with restenosis of common stents with no drug elution.

In patient-related factors, diabetes mellitus, previous bypass surgery, hypertension, number of lesions, low density lipoprotein, UA, lesion length, and left anterior descending artery stenting have been identified as risk factors for restenosis^1,16,21^. Glucose can damage arterial endothelial cells, and unbalanced glucose metabolism may activate endothelial inflammation, leading to formation of coronary atherosclerotic plaques^1,28,29^. As a risk factor for coronary heart disease, hypertension may accelerate the flow shear stress on the arterial wall, resulting in damage to the arterial endothelial cells, increased hyperplasia, and instent restenosis^30^.

Our study found that smoking was a negative risk factor for instent restenosis with an OR of 0.31, which needs further investigation. Neutrophil count was a significant risk factor for instent restenosis in our study. Increased neutrophil count may indicate elevated inflammatory reaction of the body, leading to excessive reaction of the arterial wall to the deployed stent and final restenosis.

In sex stratification analysis of risk factors for instent restenosis, QFR, smoking and neutrophil count continued to be significant predictors of restenosis in male patients. However, NLR and right coronary artery stenting were found to be new significant predictors for restenosis in males. Increased neutrophil count and NLR may indicate enhanced inflammatory reaction of the body, resulting in excessive reaction of the arterial wall to the deployed stent and final restenosis. In female patients, uric acid is a significant risk factor for restenosis, however, no independent risk factors have been detected in females. NLR has been found to be a significant factor for chronic coronary artery occlusion or instent stenosis probably because of the relationship with the high inflammation status in patients treated with PCI^31,32^. In our study, only 29 women were treated, and no significant independent risk factors were found for instent restenosis in women probably because of the small number of patients.

Some limitations existed in this study, including the retrospective and single-center study design, a small cohort of patients, Chinese patients enrolled only, no randomization, and no control, which may all influence the outcomes of this study for generalization. In addition, patients with acute coronary syndrome included in this study had a low rate of ticagrelor application after PCI and a low dose of statins, which may be associated with a higher rate of major cardiovascular adverse events. Future prospective, randomized, controlled, multicenter studies involving a large cohort of patients of multiple races and ethnicities will have to be performed for better outcomes.

In conclusion, use of sirolimus-eluting coronary stents in UA patients is associated with 17.5% rate of instent restenosis, and quantitative flow ratio after stenting, stent diameter, hypertension, and neutrophil count are significant risk factors for instent restenosis of sirolimus-coated stents in coronary intervention for patients with unstable angina.

## Data Availability

The datasets generated and/or analyzed during the current study are not publicly available due to the restriction by the hospital policy but are available from the corresponding author on reasonable request.
